# Redispersible Reduced Graphene Oxide Prepared in a Gradient Solvent System

**DOI:** 10.3390/nano12121982

**Published:** 2022-06-09

**Authors:** Yitian Sheng, Youliang Zhou, Changwei Tang, Xiangnan Cheng, Chaocan Zhang

**Affiliations:** School of Materials Science and Engineering, Wuhan University of Technology, Wuhan 430070, China; chrysemy@163.com (Y.S.); zyl_2013@whut.edu.cn (Y.Z.); tcw791880648@163.com (C.T.); chenxiangnan5523@163.com (X.C.)

**Keywords:** aggregation, surface adsorption, redispersed, gradient solvent system, conductivity

## Abstract

We designed a gradient solvent strategy for the reduction of graphene oxide, matching the hydrophilic properties of graphene oxide (GO) and reduced graphene oxide (RGO), respectively. A third solvent was added dropwise to regulate the hydrophilic variation of the continuous gradient system which maintained the whole reduction process without aggregation, and the obtained RGO dispersions could maintain stability for a long time. The separated RGO solid powder can be directly ultrasonically redispersed in N-methyl-pyrrolidone (NMP) with an average particle size as low as 200 nm. Furthermore, RGO with a high C/O ratio of 13.75 was prepared on the basis of the gradient solvent system. Using different structures of dispersants and polymers as representatives, we employed successive solvent rinsing, thermal solvent extraction, and thermal treatment to study adsorption and desorption. It was found that the above measures differed significantly in the removal of surface sorbates. The selected fatty alcohol polyoxyethylene ether (AEO) series achieved a good balance between the system dispersion and surface adsorbate removal. The conductivity was originally 5236 S m^−1^, and it increased from 9024 to 18,000 S m^−1^ after thermal treatment at 300 and 500 °C, respectively.

## 1. Introduction

GO can be obtained in large quantities as colloidal dispersions in water that are considered promising precursors for the mass production of chemically RGO [1,2,3]. RGO is widely used in making conductive films, heat-dissipation films can be spun into high-performance graphene fibers, energy storage, supercapacitors, etc., [4,5,6,7,8,9,10], due to its unique structure [11]. Numerous chemical methods for the reduction of graphene oxide have been reported [12,13,14,15,16,17]. A majority of previous studies have used hydrazine, sodium borohydride, and vitamin C (Vc) as reducing agents in aqueous or single organic solvents [18,19,20,21], where vitamin C is a natural antioxidant, and the degree of reduction is comparable to that of hydrazine reducing agents [22,23,24]. On the other hand, Hernandez et al. [25] obtained high-quality unoxidized monolayer graphene at a yield of about 1 wt.% by physical–mechanical exfoliation with dispersion and exfoliation in NMP. The authors confirmed the presence of 11 wt.% NMP residues in graphene sheets by using photoelectron spectroscopy analysis of vacuum-dried graphene sheets at room temperature. The value remained unchanged after a subsequent vacuum thermal treatment at 400 °C. The nanomaterial graphene has very strong surface activity. The surface/interface properties of graphene were reviewed by Zhao et al. [26] graphene, and its derivatives can be combined with polymers through non-covalent and covalent interactions, which had stronger interactions at the interface, tending to form extremely strong chemical bonds with polymers and even exceed chemical bonds. Wang et al. [27] investigated the difference in organic pollutant adsorption on GO and RGO to explore the potential adsorption mechanism. It was found that solution chemistry parameters affected the surface chemistry and aggregation properties of graphene, which, in turn, affected its interaction with organic contaminants. Coexisting surfactants also had different effects on the adsorption of polar and nonpolar aromatics on graphene.

Vipul Agarwal [28] reviewed the chemical reduction of graphene in solution within the last decade or so, with conductivity generally in the range of 10–7700 S m^−1^ and a C/O ratio between 3.85 and 15.1. The conductivity of graphene is mainly determined by the long-range conjugate network of the graphite lattice [29,30]. It is obvious that the chemically RGO prepared in solution is not ideal in conductivity at present. Initially, chemical reduction of graphene oxide was mostly conducted in water, where GO could be well dispersed, but the reduction product RGO could easily precipitate, leading to low reduction [31,32]. The introduction of organic weakly polar solvents aspired to minimize the generation of precipitation, and the final RGO obtained was also difficult to disperse well, due to the reduction of oxygen-containing functional groups on the surface after reduction [33]. Accordingly, we observed that the well-dispersed GO in water or a single polar organic solvent was transformed into hydrophobic RGO after reduction [34]. The change in hydrophilicity before and after reduction can easily lead to the aggregation preventing the reduction of the functional groups in the base of the layers from the reducing agent, resulting in a further incomplete reduction. It is difficult to ensure the good dispersion of both with a single reaction medium. Su et al. [35] further developed the reduction of graphene oxide in mixed solvents; however, the reduction reaction was a dynamic process, and the purely mixed solvents could not be perfectly matched with it, and some degree of aggregation might occur during the reduction process. The stable dispersion of RGO can be ensured by gradually changing the polarity of the solvent system, which facilitated a more complete reduction of graphene oxide, resulting in a good dispersion of RGO. Moreover, in terms of the unusual structure of graphene, its large π-delocalized sp^2^-bonded carbon atoms are densely arranged in planar sheets in a honeycomb lattice, giving rise to a huge specific surface area [36]. It is in parallel with the usual nanomaterials that are prone to expose obvious physical or chemical adsorption on this high-energy surface [37], which subsequently has a huge impact on the conductivity of graphene.

To deal with the three major problems of aggregation, incomplete reduction, and surface adsorption contamination in aqueous or single solvent systems, we designed a strategy of gradient solvent system. The gradual solvent system matched the hydrophobic change of the entire reduction process and the intermediate state of the GO sheet reduction structure change, thus maintaining an agglomeration-free reduction process throughout. Secondly, based on the gradual solvent system, a high concentration of reducing agents and high temperature were used to try to improve the degree of reduction. Furthermore, we used different dispersants and solvents as representatives of different types of adsorption, and then successive solvent rinsing, thermal solvent extraction, and high-temperature thermal treatment were used for the desorption study. The results showed that the reduction of GO in the gradient solvent system was without aggregation. The obtained RGO can be redispersed into NMP, with the particle size as low as about 200 nm, and the conductivity of RGO can reach 18,000 S m^−1^ after thermal treatment.

## 2. Experimental

### 2.1. Materials

Natural crystalline flake graphite (NG) (99.85% purity, 325 mesh), sulfuric acid (H_2_SO_4_, 98%), hydrochloric acid (HCl, 36%), anhydrous ethanol (≥99.7%), 2-methoxy ethanol (EGM, 99%), ascorbic acid (Vc ≥ 99%), hydrogen peroxide (H_2_O_2_, 30%), potassium permanganate (KMnO_4_), xylene, N-methyl-pyrrolidone (NMP), cyclohexane, polyvinyl pyrrolidone 30 (PVP30), polystyrene (PS), glycol phthalate (DEP), and anhydrous ethanol (C_2_H_6_O) were purchased from Sinopharm Chemical Reagent Co., Ltd. (Shanghai, China). Alkyl-phenol polyoxyethylene (7) ether (OP-7) was purchased from Wen Hua Chemical Reagent Factory (Tianjin, China), and fatty alcohol polyoxyethylene ether (AEO-3) was purchased from Jinan Maifeng Chemical Co. (Jinan, China).

### 2.2. Preparation of Graphite Oxide

GO was prepared by a modified Hummers method. The specific steps were as follows: 1 g of graphite powder was mixed with 78 mL of concentrated sulfuric acid and 12 mL of deionized water and stirred for 30 min. Then 2.5 g of potassium permanganate (KMnO_4_) was added very carefully below 5 °C. The stirring was maintained at 50 °C for 12 h. Then 100 mL of deionized water was slowly added dropwise, with vigorous stirring, below 70 °C. The mixture gradually turned into a paste and light brown solution. Subsequently, 30% H_2_O_2_ was added slowly dropwise to the mixture until no bubbles appeared. The mixture was washed by rinsing with 10% HCl and centrifugation, followed by several washes with deionized (DI) water. After filtration and vacuum drying, graphite oxide was obtained.

### 2.3. Preparation of Reduced Graphene Oxide

GO (100 mg) dissolved in EGM was mixed with different volume ratios of H_2_O, EGM or NMP, polymer, and surfactant as the initial solvent system, which was sonicated at 50 W for 2 h. The reducing agent, Vc, was added, while the third solvent was added dropwise to the above mixture. After a 4 h reaction at 80 °C, the obtained dispersion was filtered in vacuum and then rinsed with NMP 2 or 3 times. The final RGO was dried in a vacuum drying oven at 150 °C for 48 h to obtain the RGO film. RGOx changes for different conditions are indicated by the subscript x.

### 2.4. Thermal Annealing

Heat-treatment experiments were carried out in a graphite resistance furnace under a nitrogen (N_2_) atmosphere at a 2 L/min flow and heating rate of 5 °C/min. The annealing temperatures were maintained at 300 and 500 °C, respectively, for 6 h.

### 2.5. Characterization

Fourier-transform infrared (FTIR) spectra were obtained with an FTIR spectrometer (Thermo Scientific Nicolet 6700, Waltham, MA, USA). Raman spectra of graphene were obtained with Raman spectroscopy (Renishaw InVia Raman microscope, Great Britain), with a laser excitation energy of 530 nm. Particle size was obtained by laser particle sizing (Mastersizer 2000, Great Britain, UK). The morphologies and structures of GO and RGO were investigated by SEM (FESEM, Zeiss Ultra Plus, Jena, Germany), XRD (D8 Focus 3 KW, Bruker ATX, Ettlingen, Germany), and XPS (Thermo Scientific ESCALAB 250 Xi, Waltham, MA, USA). Moreover, four-point probe measurements of resistivity of RGO film were conducted by multifunction digital four-probe tester (ROOKO FT-341, Zhejiang, China).

## 3. Results and Discussion

### 3.1. Gradient Solvent Strategy

Aiming at the problem of chemical reduction of graphene oxide prone to aggregation, we designed and studied the dispersion of GO in a single solvent, mixed solvent, and gradient solvent, respectively, and established the optimal solvent selection rules.

#### 3.1.1. Determination of the Initial Solvent

GO was dispersed in different solvents and water to form a dispersion of 0.5 mg mL^−1^ and tested by laser particle size analysis. The tests showed that the dispersed binding ability of a single system of water, EGM/NMP, and xylene for GO was correlated with the solvent polarity (see Table 1). Water is currently recognized as the best good solvent for GO, as it can make GO disperse best [38,39]. GO still had good dispersion ability in the presence of polar EGM solvents. Meanwhile, NMP was generally used for the preparation and dispersion of graphene exfoliation because its surface energy was close to the exfoliation energy of the graphite sheet layer [40], and its dispersion of GO had a comparable level with EGM. Following the further weakening of the solvent polarity, GO was completely non-dispersible in xylene.

Since water has the strongest dispersing effect on GO, the reduction of GO in aqueous systems was expected to obtain a larger number and higher quality of monolayer or few-layer RGO. However, as the reduction process proceeded gradually, the insolubility of RGO in water caused severe aggregation and deteriorated the reduction environment. By cooperating with organic solvents with slightly weaker dispersing ability, it facilitated the adjustment of the dispersion state later in the reduction process. When mixed with xylene and maintained in continuous increments, the GO dispersion was at risk of aggregation, even though uniform dispersion can still be ensured within a certain range (see Table 1); however, it was unfavorable for obtaining monolayer or few-layer RGO. Meanwhile, with the addition of a small amount of water, the average particle size dropped rapidly, allowing an equivalent degree of GO dispersion to be maintained when incremental amounts of xylene were added, thus providing a greater opportunity for the dispersion of RGO in the middle and late stages of the reduction. Thus, the water/EGM, water/NMP, and water/EGM/xylene systems were able to maintain good dispersion of GO, meeting the current principles of initial solvent selection.

#### 3.1.2. Reduction of Graphene Oxide in the Gradient Solvent System

**Figure 1:**
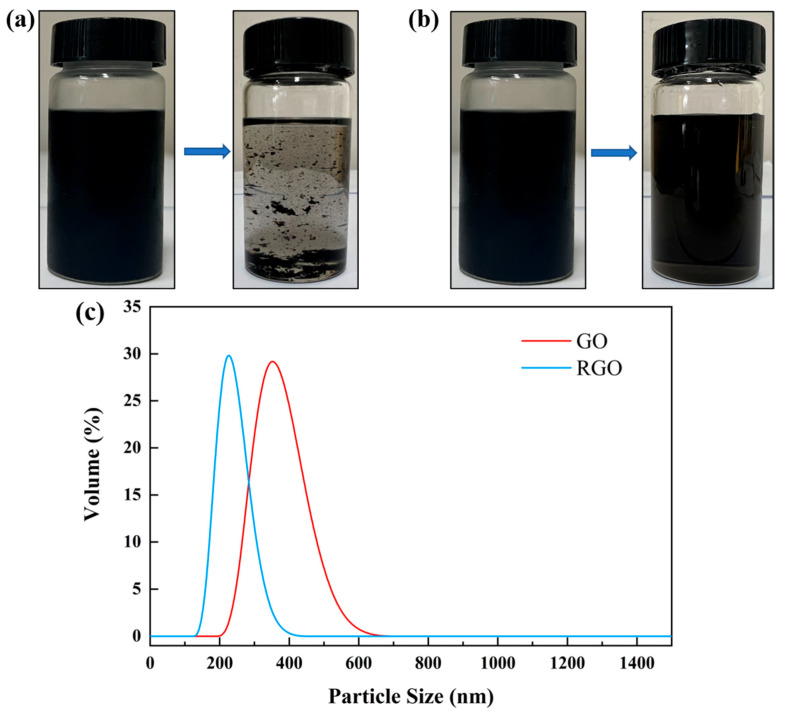
NMP dispersion of RGO before and after one month: (**a**) RGO_EGM_, (**b**) RGO_NMP_, and (**c**) redisperse RGO.

**Figure 2:**
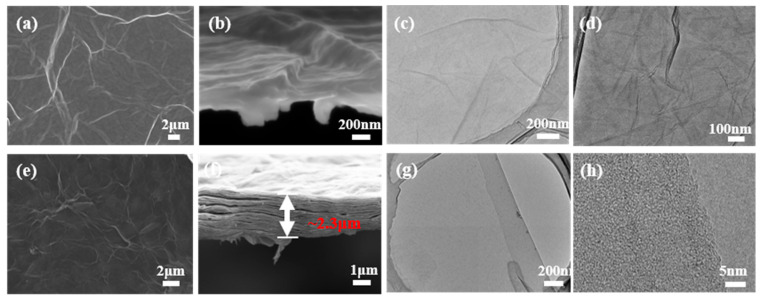
(**a**,**b**) SEM image of GO, (**c**,**d**) TEM image of GO, (**e**,**f**) SEM image of RGO, and (**g**,**h**) TEM image of RGO.

The complete matching of the GO chemical reduction process included pre-, mid-, and post-reduction and involved the regulation of a continuous hydrophilic gradient system. The reported preparation of chemically RGO by using water as a medium has resulted in the mostly poor performance of the prepared RGO due to the reaction characteristics of the ingredients, with aggregation being difficult to avoid [41,42]. We further used a gradient solvent strategy for the reduction of GO by the dropwise addition of the third solvent xylene and the intermediate solvent NMP in the water/EGM and water/NMP initial solvent systems, respectively (see Table 2). In the presence of small amounts of xylene in the water/EGM system, even with the use of NMP transition reduction intermediate, mild aggregation still occurred, comparable to that in the presence of excess xylene (nearly the same electrical conductivity). Compared with the initial system of water/NMP, the latter directly and continuously transited the pre- and mid-term solvents with the dropwise addition of progressively less polar xylenes, which made them disperse well, achieving no aggregation. The obtained NMP dispersions of RGO can remain stable (more than one month) for a long time (see Figure 1a,b), while the former dispersions showed obvious black suspensions. We separated the RGO of the above two systems to obtain the solid powder, which was then ultrasonically dispersed again. The results revealed that the RGO of water/EGM failed to disperse in NMP, while the RGO of water/NMP could be directly ultrasonically dispersed in NMP, and the average particle size of the dispersion was as low as 200 nm (Figure 1c), showing excellent redispersion performance. The folds and ripples on the GO sheet were formed by the conformation of the oxidized sp^3^ center (see Figure 2a–d). HRTEM tests at different magnifications showed that single-layer or few-layer RGO sheets could be seen clearly, and the folded edges had a uniform distribution of dark lines (see Figure 2e–h).

**Figure 3:**
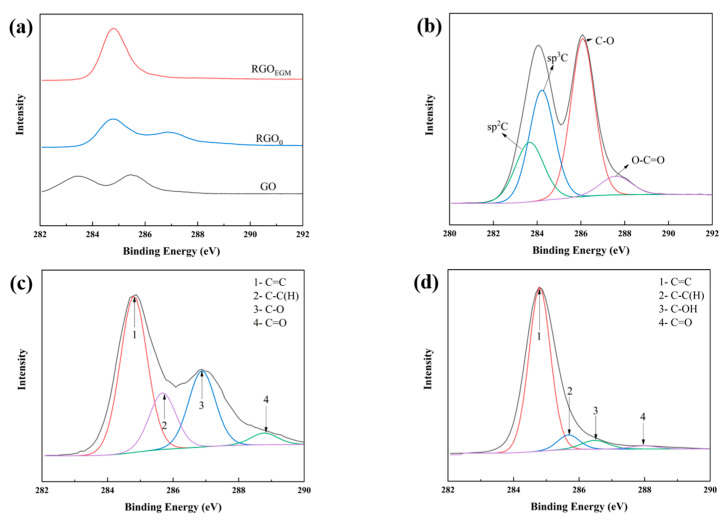
X-ray photoelectron C 1s region mapping of (**a**) GO, RGO_0_, and RGO_EGM_; C 1s X-ray photoelectron spectrum of (**b**) GO, (**c**) RGO_0_, and (**d**) RGO_EGM_.

By comparing the C1s peaks of GO and RGO in a single solvent EGM (see the last row of Table 2 for the solvent system), it was found that, although the intensity of the absorption peaks of GO was reduced in a single solvent EGM, and the intensity of the absorption peaks of the C-O and O-C=O groups was still large, in which there were still many oxygen-containing residual functional groups, and the reduction was incomplete (Figure 3b,c). However, in the gradient solvent system, the intensity of the absorption peak of sp^2^ C at the same position was greatly increased, and the intensity of the absorption peak of sp^3^ C had greatly reduced the intensity of the absorption peak of the oxygen-containing group that had been quite weak, and it even basically disappeared (Figure 3d), with a significant increase in the degree of reduction, which was also confirmed by the data of C/O ratio (Table 2 and Figure 3a). It was demonstrated that the excellent dispersion effect of this gradient solvent system can effectively avoid the occurrence of aggregation, which was consistent with the results of laser particle size.

### 3.2. Effect of Different Reducing-Agent Dosages and Reducing the Temperature on the Degree of Reduction

To address the current problem of incomplete reduction, we studied the reduction of GO with Vc as an efficient reducing agent at different temperatures and reducing-agent dosages in this paper.

In general, higher temperatures facilitate more efficient and complete chemical reactions. The ratios of the gradient solvent system were kept constant, and the GO was reduced at 80, 90, and 120 °C (labeled RGO_80_, RGO_90,_ and RGO_120_), respectively. With a higher reduction temperature, the C/O ratio of RGO decreased (see Table 3). It may be caused by the weakening of the interaction between the gradient solvent system and GO sheets at high temperatures, which made it difficult for the gradient solvent system to balance the interactions between the GO sheets, leading to slight aggregation, and was not beneficial for the continuation of the reduction, resulting the trend of decreasing reduction, which can be confirmed from the vacuum-filtration-membrane-making process. The film formation integrity of RGO_90_ began to weaken, while the RGO_120_ vacuum-filtration membrane was directly discontinuous in granular form and failed to form a film, with the dispersion showing some degree of aggregation problems. This was consistent with the trend of the C/O ratio developing.

**Figure 4:**
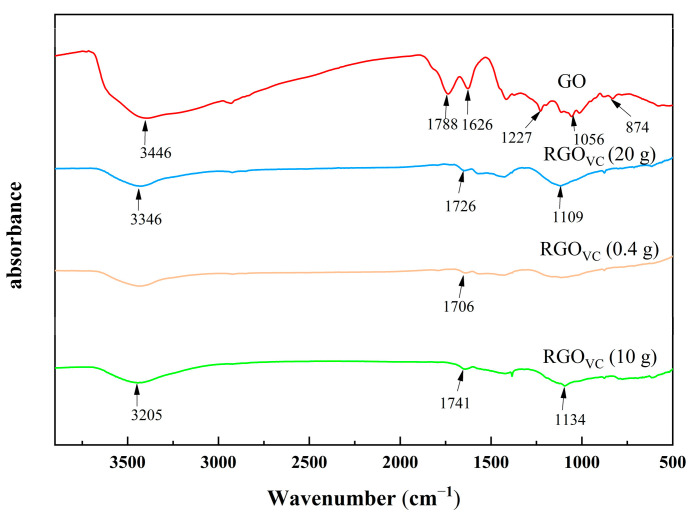
FTIR of RGO with different Vc dosages.

The ratio of the gradient solvent system was maintained, and the reductant was raised from 0.4 and 10 g to a great value of 20 g (labeled as RGO_VC10_ and RGO_VC20_, respectively). At a suitable reduction temperature (here, 80 °C), the transition time of the intermediate state was controlled to facilitate the solventization of the gradient solvent with the sheet layer. High doses of Vc were expected to completely eliminate oxygen-containing functional groups in contrast to the actual conductivity, while C/O remained almost unchanged (see Table 3). It might be that the presence of excess Vc strengthens its adsorption on the surface of the RGO sheet. From the reduction mechanism of Vc, Vc is oxidized to form dehydroascorbic acid (DHA) and two hydrogen cations. The electrophilic attack of the hydrogen cation is eliminated through further dehydration, leading to the restoration of the sp^2^ structure, and the products guluronic acids and oxalic acid have the possibility of forming hydrogen bonds with the sheet-edge carboxylic acid [43,44,45]; this strong electronic property further amplifies the adsorption of RGO, which results in a large amount of surface adsorption coating. Surface contamination from Vc obviously led to the low conductivity of RGO, while the C/O ratio was similar. The corresponding characteristic absorption peaks at 3346 and 1726 cm^−1^ confirmed the presence of Vc (see Figure 4). It proved the adsorption of Vc as a massive mask-coating mode in another way.

### 3.3. Adsorption and Desorption

Since RGO lied in a huge specific surface area, it is easy to form strong adsorption on its sheet surface. For this problem, we took the initiative to select different dispersants for dispersion and adsorption studies here. Furthermore, we adopted different strategies to remove contaminants to realize the electrical conductivity of RGO.

#### 3.3.1. Effect of Different Dispersants on Electrical Conductivity

The effects of the corresponding dispersant systems of adsorption on the conductivity are listed in Table 4 of the RGO dispersions reduced in gradient solvents, after several consecutive treatments including NMP rinsing and vacuum filtration, and then kept dry at 150 °C for 24 h. Dispersants included aromatic ring-containing, long-chain structure types such as alkyl-phenol polyoxyethylene (7) ether (OP-7), fatty alcohol polyoxyethylene ether (AEO-3), glycol phthalate (DEP), polyvinyl pyrrolidone 30 (PVP30), and polystyrene (PS). Aromatic rings and long-chain structures had better dispersion stabilization due to their strong interactions with RGO, but this strong stabilization also strengthened the adsorption influence of the system. Under the π-electron conjugation of the aromatic ring, OP-7 caused more damage to the conductivity of RGO, which was further exacerbated by long-chain types such as PVP30 and polystyrene. The conventional solvent cleaning and thermal treatment at 150 °C were not effective in removing these adsorbates. Comparative studies of these dispersants indicated that the AEO series had the lowest impact and was suitable for use as a dispersant in gradient systems.

#### 3.3.2. Thermal Extraction with Low-Boiling-Point Solvent

Referring to Hansen’s theory of solubility parameters, a solvent with a low boiling point was selected for further thermal extraction of RGO (see Table 5). The conductivity of RGO increased from ~2100 to ~3029 S m^−1^ after the first thermal reflux of EGM/cyclohexane, mainly attributed to the removal of high-boiling-point NMP. Continued secondary ethanol reflux was followed up again to ~6800 S m^−1^, which could be the removal of the mid-boiling exchange solvent EGM introduced by the first reflux transition. After drying at 150 °C, the conductivity of the first and second refluxes remained unchanged, and this was based on the direct thermal desorption of EGM (medium boiling point of 124 °C). After further drying at 300 °C, the conductivity enhancement was not obvious, and this may be attributed to the fact that the effect of solvent thermal extraction was limited to the exchange of sorbents on the surface of the RGO sheet. It was easiest to remove the multilayer adsorption on the surface of the RGO sheet at 150 °C during treatment. As the temperature continued to increase, the monolayer near the RGO surface was partially desorbed, while the activation energy required for this large amount of monolayer adsorption and the adsorption–desorption between the lamellae was difficult to be reached by conventional thermal extraction of low-boiling-point solvents, thereby exhibiting limited adsorbate removal capacity.

#### 3.3.3. Thermal Desorption

For RGO in gradient solvent systems, the use of thermal treatment to remove adsorbates upon the action of dispersant was currently one of the most effective ways. The temperature desorption of representative dispersants such as DEP, OP-7, and AEO-3 are shown in Table 6. There was an overall increasing trend in the removal of adsorbates with increasing heat-treatment temperature. Thermal treatment at 500 °C showed that AEO-3 caused weaker adsorption compared to OP-7, as is consistent with the results of multiple solvent rinses. The low conductivity of RGO without dispersant stabilization may be related to DEP series; on the other hand, due to their smaller molecular size, they were more likely to enter the interlayer of the RGO sheet, leading to adsorption, compared to the large molecule dispersants mentioned above, and needed to be removed effectively at higher temperatures, resulting in the lowest overall conductivity.

**Figure 5:**
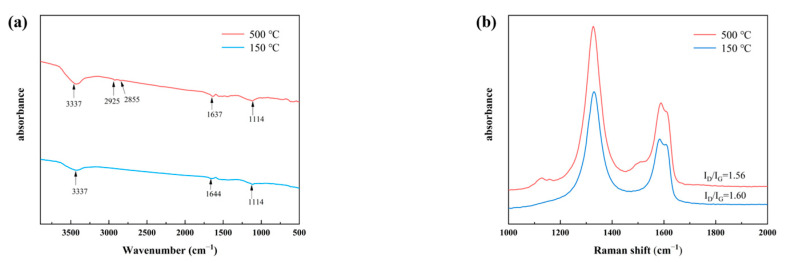
RGO heat-treated at different temperatures of (**a**) FTIR and (**b**) Raman spectra.

It had been reported in the literature that the thermal degradation temperature of various types of oxygen-containing functional groups was basically above 500 °C [46,47,48]. Thermal treatment in inert gas or vacuum does not repair the structural defects of GO remarkably below 1000 °C [49]. Hence, our selected high-temperature treatment of 500 °C mainly contributed to the removal of adsorption on the RGO surface. The Raman spectra after heat treatment showed no apparent variation in the shape, position, and intensity of the characteristic D-band and G-band peaks (see Figure 5b); the characteristic peaks of 2925 cm^−1^ and 2855 cm^−1^ for some content functional groups still existed (see Figure 5a), and these results proved that the thermal action at 500 °C did not have the ability of group elimination.

**Figure 6:**
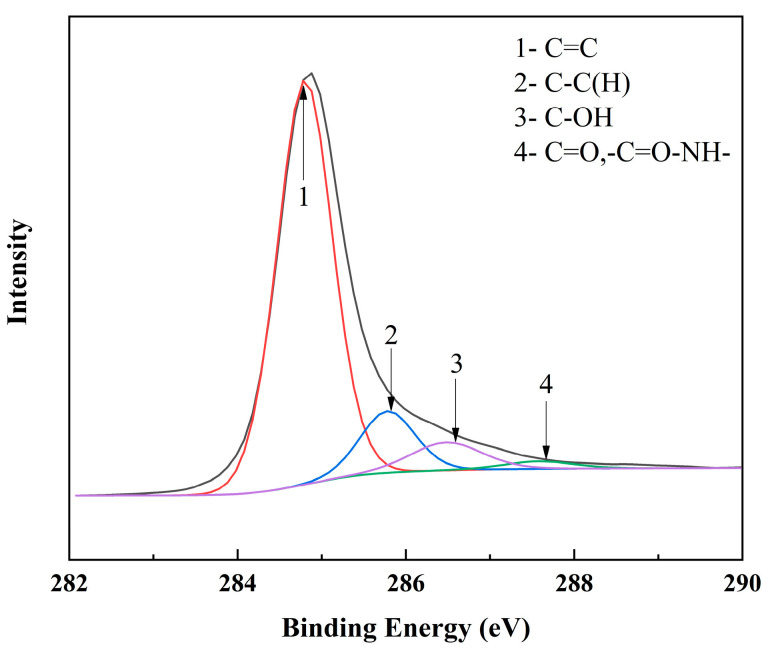
XPS of RGO heat treatment at 500 °C.

After thermal treatment at 500 °C, the functional group content of the RGO surface was confirmed by XPS (see Figure 6). The RGO of the water/NMP system showed characteristic peaks of amide bonds, owing to the presence of NMP adsorption. The main peak at 284.8 eV could be attributed to the sp^2^ carbon atom (C 1s) which formed the graphite region and demonstrated that most of the C atoms were arranged in a honeycomb lattice. The 286.48 eV component is related to the hydroxyl group (C-OH), while the 287.58 eV component is related to the carbonyl group (C = O) and the amide group (-C = O-NH) [50]. The content of the carbonyl (C = O) and amide group (-C = O-NH) was 7.13%, which proved that C, N, and O from the NMP contribution were still present in the RGO sheets, resulting in a C/O ratio exhibiting 7.92. Moreover, it provided better dispersion performance (down to 200 nm average particle size and redispersion capability) for better GO reduction. Moreover, even with partial treatment of adsorption removal, the conductivity can be increased to 18,000 S m^−1^. Consequently, the necessary adsorbate removal treatment based on the high reduction integrity of a well-dispersed system is the key to improving the conductivity of RGO.

**Figure 7:**
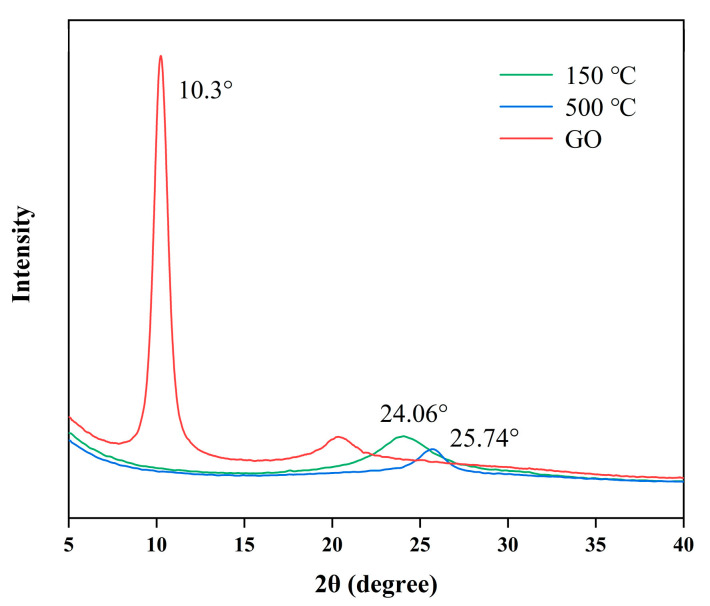
X-ray diffraction spectra of GO and RGO before and after heat treatment.

The 2θ of the 500 °C heat-treated RGO sheet increased from 24.06° to 25.74° (see Figure 7). According to the Bragg equation, higher-temperature heat treatment leads to random interlayer expansion and increased layer spacing. Volatilization of organic solvents adsorbed on the RGO surface and desorption of water between the layers led to gas generation between the layers. Cracks appeared between the graphene sheets, and the sheets became fluffy.

**Figure 8:**
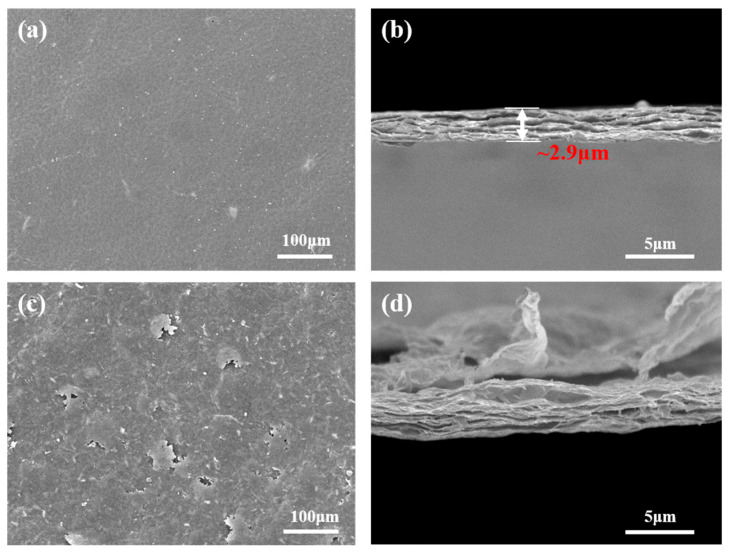
SEM images of (**a**,**b**) RGO after vacuum drying at 150 °C and (**c**,**d**) RGO after heat treatment at 500 °C.

From the microscopic morphology of the RGO sheet, the surface of the RGO sheet was rough, rippled, and folded at 150 °C drying (see Figure 8a,b). Up to 500 °C, rougher and with warped sheets of fibers, the dense accumulation of the layers was extremely spaced, possibly from the expansion caused by the escape of gas from the desorption process (see Figure 8c,d).

## 4. Conclusions

The chemical reduction of graphene oxide is a hot topic of current research in obtaining high-quality monolayers or few layers and high dispersion. Based on the analysis of a large number of reported graphene preparations, we systematically investigated the reduction-related aggregation, incomplete reduction, and surface adsorption problems to design a gradient solvent strategy. In this gradient solvent system, the incremental dropwise addition of xylene ensures the continuous dispersion of the reduction process. The prepared RGO dispersions are suitable for long-term storage and can still be redispersed to form dispersions with an average particle size as low as 200 nm, even in the dry powder state; such an excellent redispersal ability is of great advantage in the functionalization and miniaturization of graphene devices. Conventional multiple solvent washing methods, supplemented by appropriate low-temperature heat treatment, can effectively remove some of the adsorbates on the surface of RGO, resulting in a marked increase in conductivity to 18,000 S m^−1^. This has provided a feasible direction for the preparation of highly conductive chemically reduced graphene, with the choice of lower-boiling-point solvents and dispersants of less conjugated structure contributing more readily to the high conductivity properties during reduction.

## Figures and Tables

**Table 1:** Dispersion of GO in single/mixed-solvent system.

H_2_O (mL)	EGM (mL)	NMP (mL)	Xylene (mL)	Average Particle Size (nm)
14	/	/	/	278 ± 20
/	14	/	/	594 ± 20
/	/	/	14	Non-diversified
/	12	/	2	963 ± 20
/	10	/	4	1604 ± 20
/	8	/	6	Flocculation
1	12	/	2	628 ± 20

**Table 2:** RGO in the gradient solvent system.

Initial Solvent			Mid-Term Solvent	Post-Solvent	Dispersion	C/O Ratio	Electrical Conductivity (S m^−1^)
H_2_O (mL)	EGM (mL)	NMP (mL)	NMP (mL)	Xylene/mL			
10	90	/	/	40	Uniform dispersion	13.54 ^a^	4363 ± 300
10	60	/	80	20	Slight aggregation	/	3745 ± 300
10	60	/	60	40	Uniform dispersion	9.60 ^b^	4014 ± 300
10	60	/	40	60	Uniform dispersion	/	3948 ± 300
10	60	/	20	80	Slight aggregation	/	3508 ± 300
10	/	90	/	40	Uniform dispersion	7.92 ^c^	5236 ± 300
0	100	/	/	0	Aggregation	3.09 ^d^	124 ± 300

^a^ RGO_EGM_. ^b^ RGO. ^c^ RGO_NMP_. ^d^ RGO_0_.

**Table 3:** RGO at different temperatures and dosages of reducing agents in the gradient solvent system.

Sample Number	Temperature (°C)	Vc Dosage (g)	C:O Ratio ^a^	Electrical Conductivity (S m^−1^)	RGO Dispersions
RGO_80_	80	0.4	7.92	5236	Uniform dispersion
RGO_90_	90	0.4	7.20	3190	Slight aggregation
RGO_120_	120	0.4	6.15	—	Aggregation
RGO_VC10_	80	10	7.58	1284	Uniform dispersion, stable
RGO_VC20_	80	20	7.39	1268	Uniform dispersion, stable

^a^ Results from XPS.

**Table 4:** Conductivity of RGO with different dispersants.

Dispersant	OP-7	AEO-3	DEP	PVP30	PS
Conductivity (S m^−1^)	4679	5236	1000	125	898

**Table 5:** The conductivity of thermally extracted RGO.

Sample Number ^a^	Conductivity (S m^−1^)		
	Vacuum Filtration	First Thermal Reflux	Second Thermal Reflux
RGO_T70_	2100	3029	6800
RGO_T150_	5236	6209	6580
RGO_T300_	9024	9130	9200

^a^ AEO-3 was used as a dispersant.

**Table 6:** The conductivity of RGO with different temperature heat treatments.

Heat Treatment Temperature (°C)	Conductivity (S m^−1^)			
	AEO-3	OP-7	DEP	/
70	2100	1056	260	700
150	5236	4083	1000	2401
300	9024	8771	2034	8425
500	18,000	12,000	2984	8425

## Data Availability

The data presented in this study are available upon request from the corresponding author.
